# GalaxyPepDock: a protein–peptide docking tool based on interaction similarity and energy optimization

**DOI:** 10.1093/nar/gkv495

**Published:** 2015-05-12

**Authors:** Hasup Lee, Lim Heo, Myeong Sup Lee, Chaok Seok

**Affiliations:** 1 Department of Chemistry, Seoul National University, Seoul 151–747, Korea; 2 Department of Biomedical Sciences, College of Medicine, University of Ulsan, Seoul 138–736, Korea

## Abstract

Protein–peptide interactions are involved in a wide range of biological processes and are attractive targets for therapeutic purposes because of their small interfaces. Therefore, effective protein–peptide docking techniques can provide the basis for potential therapeutic applications by enabling an atomic-level understanding of protein interactions. With the increasing number of protein–peptide structures deposited in the protein data bank, the prediction accuracy of protein-peptide docking can be enhanced by utilizing the information provided by the database. The GalaxyPepDock web server, which is freely accessible at http://galaxy.seoklab.org/pepdock, performs similarity-based docking by finding templates from the database of experimentally determined structures and building models using energy-based optimization that allows for structural flexibility. The server can therefore effectively model the structural differences between the template and target protein–peptide complexes. The performance of GalaxyPepDock is superior to those of the other currently available web servers when tested on the PeptiDB set and on recently released complex structures. When tested on the CAPRI target 67, GalaxyPepDock generates models that are more accurate than the best server models submitted during the CAPRI blind prediction experiment.

## INTRODUCTION

Protein–protein interactions that are mediated by short linear peptides of interacting partners are critical in a broad range of biological processes, such as signaling pathways, protein cellular localization and post-translational modifications (1–4). The importance of such interactions is evident because of their involvement in critical human diseases, such as cancer and infections (5,6). Because of the small sizes of protein–peptide interfaces, such interactions can be modulated by small chemicals or synthetic peptides (7,8). Therefore, effective computational modeling of protein–peptide interactions can provide useful information for understanding complex biological processes in molecular detail and for modulating protein–protein interactions for disease treatment.

As in other areas of molecular modeling, it is very difficult to obtain reliable predictions by computational protein–peptide docking when prior knowledge of the interactions is not available. When there is no information on the binding site, putative binding sites must be searched for on the entire surface of the target protein. Such global docking methods show limited accuracy for predicting high-resolution complex structures, but successful predictions of at least part of the binding residues have been reported (9–11). When experimental or predicted data on binding site residues are available, such information can be used to constrain the docking to local regions of the protein surface (12). These local docking methods usually require a model protein–peptide complex structure as input, whereas global docking methods require only a protein structure and a peptide sequence. Among the various protein–peptide docking methods developed so far, only a small number of methods are available as web servers, such as PepSite (13) and PEP-SiteFinder (14) for global docking and Rosetta FlexPepDock (15–17) and PepCrawler (18) for local docking.

As increasing number of protein–peptide complex structures are being deposited in the protein data bank, the probability of finding protein–peptide complexes similar to a given target complex in the structure database increases. For example, 87% of the non-redundant protein–peptide complexes in the PeptiDB set (19) have similar proteins, with a protein TM-score > 0.6, among the experimentally resolved structures that were published prior to the given complex. Because protein–peptide interactions are usually stabilized through hot spot interactions (19,20), the observed hot spot interactions in known protein–peptide complex structures can be useful for predicting interactions that involve a range of new variations in target proteins and peptides.

The GalaxyPepDock server presented in this paper utilizes information on protein–peptide interactions of similar proteins in the database of experimentally determined structures to generate high-resolution complex structures when reasonable template protein–peptide complex structures can be found. A further refinement by GALAXY energy-based optimization (21–24) enables the modeling of structural differences between the template and target complex structures by sampling the backbone and side-chain flexibilities of both protein and peptide.

GalaxyPepDock identified 75.4% of the binding site residues on average, compared with 66.2 and 40.9% by PEP-SiteFinder (14) and PepSite (13), respectively, for the 40 PeptiDB targets that have ≤ 10 residue-long peptides that are accepted by PepSite. In terms of complex structure prediction, GalaxyPepDock returned structures with better than acceptable quality when measured by the CAPRI criterion ((25), http://www.ebi.ac.uk/msd-srv/capri/round28/round28.html) for 37 of the 57 PeptiDB targets, compared with the 9 targets returned by PEP-SiteFinder. A similar level of improvement by GalaxyPepDock was also observed when tested on 22 recently released protein–peptide complex structures. When tested on the CAPRI target 67, predictions of medium accuracy were made; this accuracy is among the best predictions made by human groups and superior to the best server predictions submitted during the CAPRI blind prediction experiment. For this target, the conformational change of the protein by peptide binding was also correctly predicted.

## THE GalaxyPepDock METHOD

**Figure 1.**
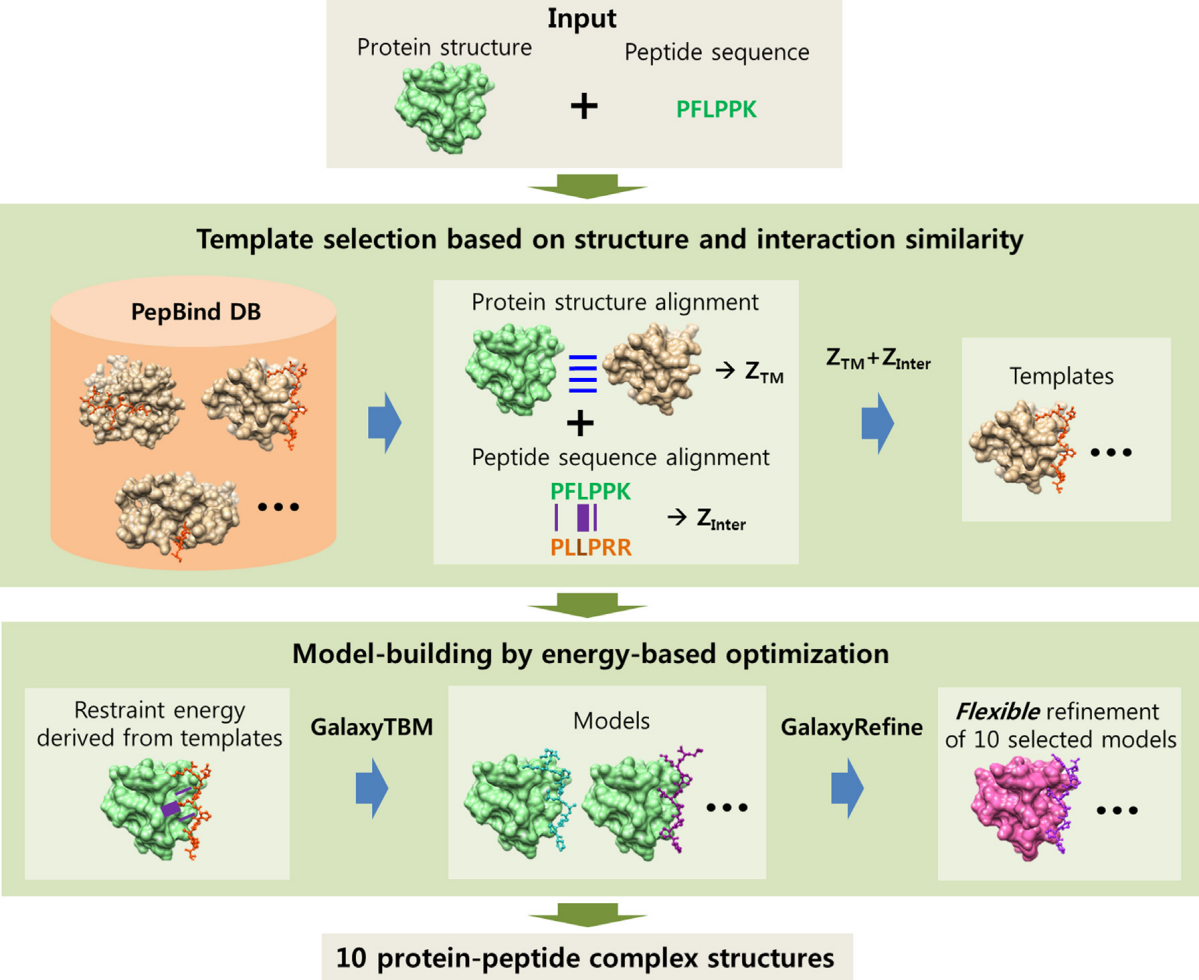
Flowchart of the GalaxyPepDock algorithm. Given a protein structure and a peptide sequence, template complex structures are first selected from the PepBind database based on protein structure similarity and protein–peptide interaction similarity. Models are then built with the model-building tool of GalaxyTBM, and the 10 models that are selected based on energy are returned after further optimization by the GalaxyRefine flexible refinement method.

The overall procedure of template selection and model-building is illustrated in Figure 1 and is described in more detail below.

### Template selection

Templates for protein–peptide complex structure prediction are selected from the PepBind (26) database with the following score for each complex structure in the database

\begin{equation*} S_{{\rm complex}} = Z_{{\rm TM}} + Z_{{\rm Inter}} , \end{equation*}

where *Z*_TM_ measures the protein structure similarity by the *Z*-score of the TM-score of a database protein structure when aligned to the target protein structure by TM-align (27) and *Z*_Inter_ measures the interaction similarity of a database complex and the target complex when aligned to the former by the *Z*-score of the interaction similarity score *S*_Inter_ defined below. Up to 10 complexes with *S*_complex_ > 90% of the maximum value are selected as templates and used in the model-building procedure described in the next subsection.

To measure the interaction similarity of a database complex and the target complex, the target complex is first aligned to the database complex by protein structure alignment and peptide sequence alignment. Peptide alignment is performed by gapless sequence alignment with a modified BLOSUM62 (28) matrix score, by multiplying the weight of (1 + the number of hydrophobic or ionic protein residues contacting the given peptide residue in the template complex structure) to the BLOSUM62 matrix components with scores > 0. Hydrophobic (or ionic) protein–peptide residue pairs with at least one heavy atom pair within 5.0 Å (or 6.0 Å) are considered to be contacting following the PepBind criterion (26). In this way, more emphasis is put on the peptide residues contributing to hot spot interactions than on other residues during peptide alignment. An example case of peptide alignment is provided in Supplementary Figure S1(a). The interaction similarity score *S*_Inter_ is then calculated by summing the interaction pair similarity score $S_{i - j}$ for all of the protein–peptide residue pairs $(i - j)$ in contact in the template complex, as illustrated in Supplementary Figure S1 (b) for the example case. $S_{i - j}$ is measured by the similarities in the amino acids of the contacting pair $(i - j)$ in the template complex and of the corresponding pair $(i^\prime - j^\prime )$ in the target complex aligned to the template and is defined as $S_{i - j} = {\rm Max}\left[ {B(i,i^\prime ) + B(j,j^\prime ),B(i,j^\prime ) + B(j,i^\prime )} \right]$, where $B(i,i^\prime )$ is the BLOSUM62 matrix component for the amino acid of residue $i$ and that of residue $i^\prime$.

### Model-building

For each template, 50 model complex structures are first generated with the model-building tool of GalaxyTBM (29,30) using protein structure alignment and peptide sequence alignment. For the model-building optimization, restraints on the distances between interacting protein–peptide pairs are added to the GALAXY energy, with weights dependent on the interaction pair similarity score $S_{i - j}$ (see Supplementary Figure S1(c) for details). Interaction pairs with higher similarities to the template tend to be conserved by stronger template-derived restraints, whereas the sampling of other parts of the structure is driven more by the physics-based energy than by template-derived information. Of the model structures generated by GalaxyTBM, 10 structures are selected by choosing the structures with the best energy values for each template and are further refined following the GalaxyRefine (21) protocol. This refinement step allows for the adjustment of the backbone and side-chain structures by repetitive molecular dynamics relaxations after side-chain repacking.

### Performance of the method

The performance of GalaxyPepDock was compared with those of two available web servers, PEP-SiteFinder and PepSite, which perform global docking and thus do not require the protein–peptide structure as input. Because PEP-SiteFinder and PepSite are *ab initio* methods that do not rely on template information, the comparison of the results presented here demonstrate the extent to which a similarity-based method such as GalaxyPepDock can be useful compared with the *ab initio* methods for the benchmarking set. For a fair comparison, the complexes in the PepBind database that were released after each target complex were excluded during template search in GalaxyPepDock prediction. The accuracy of the best model of the 10 generated models was evaluated for each method.

The non-redundant set of PeptiDB (19) was first employed for comparison. Peptide docking to unbound protein structures was performed on 57 of the 103 PeptiDB complexes for which unbound protein structures are available in the structure database because re-docking peptides to bound protein structures is only of theoretical interest. For the 40 PeptiDB targets that have ≤ 10 residue-long peptides that are accepted by PepSite, GalaxyPepDock identified 75.4% of the binding site residues on average, compared with the 66.2% and 40.9% identified by PEP-SiteFinder and PepSite, respectively (see Supplementary Table S1 for results for individual targets). In terms of complex structure prediction, GalaxyPepDock generated structures with better than acceptable quality when measured by the CAPRI criterion (25, http://www.ebi.ac.uk/msd-srv/capri/round28/round28.html) for 37 of the 57 PeptiDB targets, compared with the 9 targets returned by PEP-SiteFinder (see Supplementary Tables S2 and S3 for details).

It should be emphasized that the flexible-structure energy-based model-building procedure of GalaxyPepDock can improve the predictions beyond that of a simple method that superimpose the target onto the template structure. The improvement in prediction accuracy achieved by additional energy optimization compared with the template superimposition method can be observed from the increased number of high-accuracy/medium-accuracy/acceptable predictions from 5/22/36 to 6/27/37 and the improved median ligand-RMSD/interface-RMSD/(fraction of native contact) values from 3.0 Å/1.6 Å/0.571 to 2.8 Å/1.3 Å/0.667.

For an independent test, protein–peptide complexes involving 5- to 15-residue peptides and with available unbound protein structures that share ≤70% sequence identity were selected among recently released structures (released after 2009). This set consists of 22 complexes. On this set, GalaxyPepDock showed a similar level of improvement, producing structures with better than acceptable quality for 17 of the 22 targets, compared with 5 returned by PEP-SiteFinder (see Supplementary Tables S4 and S5 for details). The number of high-accuracy/medium-accuracy/acceptable predictions was increased from 0/8/15 to 0/9/17 and median ligand-RMSD/interface-RMSD/(fraction of native contact) values were improved from 3.1 Å/1.8 Å/0.588 to 2.5 Å/1.6 Å/0.693 by energy optimization.

GalaxyPepDock was also tested on the CAPRI target 67 (PDB ID: 4N7H), and a medium-accuracy prediction was made (see Supplementary Table S6). Compared with template-superimposed models, the quality of the model was improved by energy optimization from acceptable to medium accuracy, with improvements in ligand-RMSD/interface-RMSD/(fraction of native contact) values from 2.9 Å/1.5 Å/0.500 to 1.8 Å/1.0 Å/0.688. In the CAPRI blind prediction experiment, no group submitted high-accuracy models for this target, and 6 of the 44 registered groups submitted medium-accuracy models. The best server predictions were only of acceptable quality. The ‘Seok’ group submitted models only of acceptable quality because no refinement procedure was applied at the time.

## THE GalaxyPepDock SERVER

### Hardware and software

The GalaxyPepDock server runs on a cluster of 12 Linux servers of 2.33-GHz Intel Xeon 8-core processors. The web application uses the Python programming language and the MySQL database. The protein–peptide docking pipeline is implemented using Python. The protein–peptide docking algorithm is implemented in the GALAXY program package (29,31) written in Fortran 90. The JavaScript Protein Viewer (http://biasmv.github.io/pv/) is used for the visualization of the predicted models.

### Input and output

**Figure 2.**
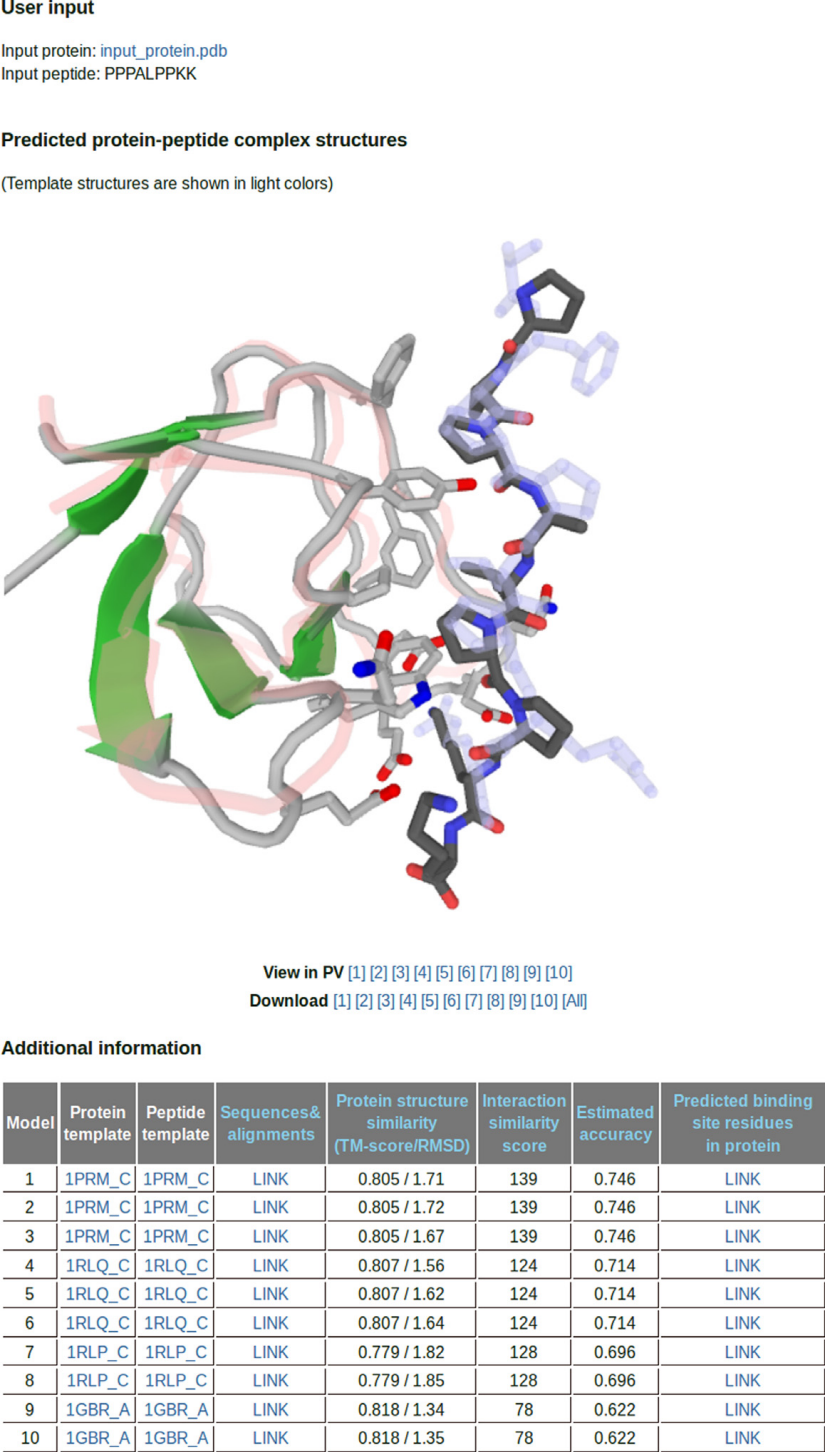
GalaxyPepDock output page. Generated models are shown in the images and can be downloaded or viewed using the JavaScript Protein Viewer. Additional information such as selected templates, alignments of query and template sequences, protein structure similarity, interaction similarity score, estimated accuracy and predicted binding site residues is also provided.

The required input is a protein structure in PDB format and a peptide sequence in FASTA format. The size of the target protein and peptide is limited to 900 and 30 amino acids, respectively, for computational efficiency. The average run time is 2–3 h. Ten model structures can be viewed and downloaded from the website, and additional information on the predicted binding sites and estimated accuracy of the predicted interactions is provided. The prediction accuracy is estimated from a linear model of the relationship between the fraction of correctly predicted binding site residues and the template-target similarity (measured by the protein TM-score and *S*_Inter_) obtained by a linear regression of the PeptiDB test set results. For targets with a low estimated accuracy close to 0, it is suggested that *ab initio* docking servers such as PEP-SiteFinder are tried. A sample output page is shown in Figure 2.

## CONCLUSIONS

GalaxyPepDock is a similarity-based protein–peptide docking web server that performs additional flexible-structure energy-based optimization. The effective combination of database search and physics-based optimization allows for a superior performance compared with the existing methods when complexes involving similar proteins can be found in the database.

## SUPPLEMENTARY DATA

Supplementary Data are available at NAR Online.

SUPPLEMENTARY DATA
