# Supplementary material for: GalaxyPepDock: a protein–peptide docking tool based on interaction similarity and energy optimization
Source: Nucleic Acids Res. 2015 May 12;43(Web Server issue):W431–5. doi: 10.1093/nar/gkv495 (PMC4489314; doi:10.1093/nar/gkv495)
Supplement: SUPPLEMENTARY DATA [file supp_43_W1_W431__index.html]

GalaxyPepDock: a protein–peptide docking tool based on interaction similarity and energy optimization — GalaxyPepDock: a protein–peptide docking tool based on interaction similarity and energy optimization — SUPPLEMENTARY DATA 

# GalaxyPepDock: a protein–peptide docking tool based on interaction similarity and energy optimization

## SUPPLEMENTARY DATA

- SUPPLEMENTARY DATA
