# Supplementary material for: GalaxyPepDock: a protein–peptide docking tool based on interaction similarity and energy optimization
Source: Nucleic Acids Res. 2015 May 12;43(Web Server issue):W431–5. doi: 10.1093/nar/gkv495 (PMC4489314; doi:10.1093/nar/gkv495)
Supplement: SUPPLEMENTARY DATA [file supp_gkv495_nar-00411-web-b-2015-File004.pdf]

Figure S1. The procedures of peptide alignment, calculation of interaction similarity score, and construction of the energy function for model building optimization illustrated for an example case.

(a) Peptide alignment performed with a modified BLOSUM62 matrix

j' =		1	2	3	4	5	6	7	8	
Query peptide	—	P	P	P	A	L	P	P	K	K
		|	|	|	|	|	|	|	|	
Template peptide	A	F	A	P	P	L	P	R	R	—
j =		1	2	3	4	5	6	7	8	

Scores

B(j , j')[1]	-8	-4	-1	7	-2	4	7	-2	2	-8
N_{inter}(j)[2]	0	1	2	2	1	4	3	0	1	—
Modified B(j , j')[3]	-8	-4	-1	21	-2	20	28	-2	4	-8

[1]B(j , j') = BLOSUM62[amino acid (j), amino acid (j')]

[2]N_{inter}(j) = Number of interacting hydrophobic or ionic protein residues for peptide residue j in the template complex structure

[3]Modified B(j , j') = [1+N_{inter}(j) x Θ{B(j, j')}] x B(j , j')

Θ(B) = 0 if B ≤ 0, 1 if B > 0

(b) Calculation of interaction similarity score S_{Inter}

j' =	...	3	4	5	6	...
Query peptide	- - -	P	A	L	P	- - -
		|	|	|	|	
Template peptide	- - -	P	P	L	P	- - -
j =	...	3	4	5	6	...

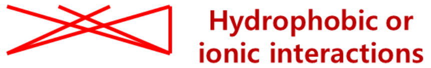

i =	...	49	50	51	52	...
Template protein	- - -	P	S	N	Y	- - -
		|	|	|	|	
Query protein	- - -	P	V	P	Y	- - -
i' =	...	49	50	51	52	...

$$S_{\text{Inter}} = \sum_{\text{all } (i,j) \text{ pairs}} S_{i-j}$$

$$S_{i-j} = Max\left[B(i,i') + B(j,j'), B(i,j') + B(j,i')\right]$$

(i,j)	(49,5)	(49,6)	(52,3)	(52,4)	(52,6)	...
S_{i-j}	11	14	14	6	14	...

(c) Energy for model building optimization

$$E\left(\left\{\mathbf{r}_{i'},\mathbf{r}_{j'}\right\}\right)=E_{\mathrm{GALAXY}}\left(\left\{\mathbf{r}_{i'},\mathbf{r}_{j'}\right\}\right)+\sum_{\text{all }(i,j)\text{ pairs}} k_{i-j}\left(r_{i'j'}-r_{i'j'}^{(0)}\right)^2$$

(i,j)	(49,5)	(49,6)	(52,3)	(52,4)	(52,6)	...
S_{i-j}	11	14	14	6	14	...
k_{i-j}	6	6	6	4	6	...

S_{i-j}	Weight (k_{i-j})
$S_{i\text{-}j} < 0$	1
$0 < S_{i\text{-}j} \leq 5$	2
$5 < S_{i\text{-}j} \leq 10$	4
$10 < S_{i\text{-}j}$	6

Table S1. Fraction of binding site residues correctly predicted by GalaxyPepDock, PEP-SiteFinder, and PepSite on the 40 targets of the PeptiDB set that have available unbound protein structures and have ≤ 10 residue-long peptides.

PDB ID		Galaxy PepDock	PEP-Site Finder	PepSite
Bound	Unbound			
1ER8_E:I	1OEW_A	0.969	0.813	0.313
1CKA_A:B	2DVJ_A	0.800	0.867	0.733
1AWR_C:I	2ALF_A	1.000	0.813	0.750
1CZY_C:E	1CZZ_C	1.000	0.000	0.000
1DDV_A:B	1I2H_A	0.900	0.500	0.000
1H6W_A:B	1OCY_A	0.742	0.742	0.677
1KL3_C:G	2RTM_A	1.000	0.647	0.000
1GYB_B:E	1GY7_B	0.125	0.250	0.250
1LVM_A:E	1LVB_B	0.000	0.385	0.000
1MFG_A:B	2H3L_A	1.000	0.941	0.647
1N7F_B:D	1N7E_A	0.938	0.875	0.000
1OAI_A:B	1GO5_A	0.200	0.933	0.333
1NVR_A:B	2QHN_A	0.000	1.000	1.000
1OU8_B:D	1OU9_A	0.000	0.810	0.000
1UJ0_A:B	1X2Q_A	0.933	0.733	0.933
1T4F_M:P	1Z1M_A	0.824	0.647	0.353
1T7R_A:B	2AM9_A	0.938	0.875	0.000
1VZQ_H:I	1JWT_A	1.000	0.231	0.000
1TP5_A:B	1PDR_A	1.000	0.722	0.944
1W9E_A:T	1R6J_A	0.875	1.000	0.000
1YWO_A:P	1Y0M_A	1.000	1.000	1.000
1X2R_A:B	1X2J_A	0.818	0.727	0.773
2AK5_B:D	2G6F_X	1.000	0.833	1.000
2B1Z_B:D	3ERT_A	0.692	0.000	0.000
2C3I_B:A	2J2I_B	0.905	0.952	0.000
2FGR_A:B	2FGQ_X	0.900	0.300	0.000
2FOJ_A:B	2F1W_A	0.867	0.800	0.667
2FVJ_A:B	2HWQ_A	1.000	0.933	0.400
2H9M_C:D	2H14_A	0.900	0.800	0.800
2DS8_B:P	2DS7_A	0.538	0.077	0.385
2HO2_A:B	2E45_A	0.875	1.000	1.000
2HPL_A:B	2HPJ_A	0.000	0.929	0.500
2O9V_A:B	2O9S_A	1.000	0.750	0.833
2P1T_A:B	1LBD_A	0.588	0.647	0.118
2PUY_B:E	2YQL_A	1.000	0.889	0.500
2R7G_C:D	1AD6_A	0.895	0.789	0.211
2VJ0_A:P	1B9K_A	0.643	0.143	0.643
2ZJD_A:B	1V49_A	0.750	0.500	0.000
3D1E_A:P	3D1G_A	0.818	0.500	0.545
3D9T_B:D	1QBH_A	0.714	0.143	0.071
Average		0.754	0.662	0.409
Median		0.885	0.760	0.369

Table S2. Ligand-RMSD (LRMSD), interface-RMSD (IRMSD), fraction of native contact (fnat), and model quality measured by the CAPRI criterion (***/**/* for high-accuracy/medium-accuracy/acceptable prediction) for the predictions made on the 57 targets of the PeptiDB set that have available unbound structures by GalaxyPepDock and PEP-SiteFinder.

PDB_ID		GalaxyPepDock				PEP-SiteFinder			
Bound	Unbound	LRMSD	IRMSD	fnat	Quality	LRMSD	IRMSD	fnat	Quality
1ER8_E:I	1OEW_A	0.84	0.42	0.949	***	7.40	2.91	0.205	-
1CKA_A:B	2DVJ_A	2.80	1.22	0.500	*	5.76	2.69	0.409	-
1AWR_C:I	2ALF_A	1.37	0.59	0.800	**	5.80	2.64	0.280	-
1SFI_A:I	1UTN_A	2.81	1.24	0.769	*	9.62	5.57	0.077	-
1CZY_C:E	1CZZ_C	1.04	0.42	0.923	***	29.52	12.06	0.000	-
1DDV_A:B	1I2H_A	7.23	2.67	0.429	-	11.16	4.82	0.214	-
1EG4_A:P	1EG3_A	42.96	18.15	0.000	-	16.67	8.83	0.048	-
1JBU_H:X	2BZ6_H	26.03	12.87	0.026	-	4.85	2.99	0.342	-
1H6W_A:B	1OCY_A	13.92	6.07	0.250	-	12.36	5.18	0.146	-
1KL3_C:G	2RTM_A	3.69	1.88	0.632	*	8.66	3.83	0.105	-
1GYB_B:E	1GY7_B	27.02	7.60	0.000	-	15.45	4.56	0.100	-
1LVM_A:E	1LVB_B	26.44	10.51	0.000	-	18.06	7.03	0.000	-
1MFG_A:B	2H3L_A	2.68	1.42	0.714	*	13.92	7.26	0.048	-
1N7F_B:D	1N7E_A	1.23	0.65	0.808	**	3.17	1.44	0.577	*
1OAI_A:B	1GO5_A	23.36	8.85	0.091	-	4.64	2.05	0.409	-
1NVR_A:B	2QHN_A	28.90	10.91	0.000	-	5.48	2.74	0.667	-
1NX1_A:C	1ALV_A	19.25	9.10	0.125	-	34.17	14.50	0.000	-
1OU8_B:D	1OU9_A	25.47	11.39	0.000	-	4.16	2.33	0.267	-
1UJ0_A:B	1X2Q_A	1.00	0.69	0.600	**	7.70	3.57	0.450	-
1RXZ_A:B	1RWZ_A	1.71	1.45	0.788	**	8.07	3.99	0.212	-
1SSH_A:B	1OOT_A	1.38	0.59	0.800	**	5.32	2.80	0.400	-
1T4F_M:P	1Z1M_A	1.30	1.29	0.696	**	5.50	2.91	0.261	-
1T7R_A:B	2AM9_A	1.13	0.66	0.727	**	2.81	1.06	0.500	*
1VZQ_H:I	1JWT_A	1.37	0.69	0.944	**	13.83	6.56	0.056	-
1TP5_A:B	1PDR_A	0.97	0.58	0.773	**	6.48	2.44	0.318	-
1W9E_A:T	1R6J_A	0.79	0.43	1.000	***	5.18	2.05	0.421	-
1YUC_A:C	1PK5_A	2.03	0.89	0.720	**	34.37	13.70	0.000	-
1YWO_A:P	1Y0M_A	3.54	0.79	0.643	**	4.83	2.35	0.286	-
1X2R_A:B	1X2J_A	8.18	3.87	0.222	-	6.39	2.75	0.185	-
2A3I_A:B	2AA2_A	2.99	0.29	0.833	***	1.83	0.38	0.722	**
2AK5_B:D	2G6F_X	1.19	0.75	0.800	**	11.84	4.74	0.267	-
2B1Z_B:D	3ERT_A	4.78	4.29	0.333	-	29.36	11.98	0.000	-
2C3I_B:A	2J2I_B	0.80	0.45	0.852	**	4.74	2.62	0.370	-
2B9H_A:C	2B9F_A	4.79	2.22	0.414	-	5.91	2.91	0.207	-
2FGR_A:B	2FGQ_X	1.49	0.87	0.950	**	16.75	7.54	0.050	-
2FMF_A:B	1JBE_A	1.31	0.62	0.867	**	2.43	0.77	0.733	**
2FOJ_A:B	2F1W_A	4.54	2.17	0.429	-	5.84	2.78	0.333	-
2CCH_D:F	1H1R_B	1.46	0.76	0.781	**	4.50	2.82	0.250	-
2FVJ_A:B	2HWQ_A	0.96	0.36	0.789	**	1.77	0.56	0.684	**
2H9M_C:D	2H14_A	0.94	0.51	0.842	***	4.16	1.71	0.421	*
2DS8_B:P	2DS7_A	14.16	5.51	0.154	-	16.42	5.94	0.000	-
2HO2_A:B	2E45_A	16.73	5.30	0.267	-	4.44	0.58	0.800	**
2HPL_A:B	2HPJ_A	41.79	17.32	0.000	-	3.42	1.96	0.737	*
2O02_A:P	2BQ0_A	2.06	1.29	0.765	*	13.06	6.98	0.059	-
2O4J_A:C	1IE9_A	0.91	0.43	0.870	***	23.95	9.57	0.000	-
2O9V_A:B	2O9S_A	1.16	0.55	0.813	**	6.00	2.01	0.375	-
2P1K_A:C	1PWJ_A	3.31	3.31	0.455	*	18.11	8.17	0.061	-
2P1T_A:B	1LBD_A	1.32	10.31	0.536	**	13.94	6.31	0.214	-
2P54_A:B	1I7G_A	1.98	0.70	0.750	**	20.93	8.97	0.000	-

2PUY_B:E	2YQL_A	3.08	1.21	0.741	*	14.43	6.38	0.111	-
2QOS_C:A	1LF7_A	6.20	2.81	0.200	-	5.45	2.36	0.250	-
2R7G_C:D	1AD6_A	3.49	1.87	0.714	*	3.07	1.81	0.476	*
2VJ0_A:P	1B9K_A	10.88	4.95	0.143	-	26.04	10.37	0.095	-
3BU3_A:B	1P14_A	4.44	1.45	0.444	*	22.74	8.85	0.000	-
2ZJD_A:B	1V49_A	8.32	3.67	0.214	-	7.97	3.63	0.214	-
3D1E_A:P	3D1G_A	1.58	0.79	0.667	**	9.49	4.03	0.185	-
3D9T_B:D	1QBH_A	3.70	1.91	0.526	*	16.74	6.27	0.000	-
Average		7.56	3.41	0.545		10.99	4.75	0.256	
Median		2.81	1.29	0.667		7.70	3.57	0.214	

Table S3. Similarity of the query and the template protein structures measured by TM-score, sequences of the query and the template peptides and ligand RMSD of the starting complex and the final model on the 57 targets of the PeptiDB set.

ID (Query)	ID (Template)	TM-Score	Query peptide	Template peptide	Initial RMSD	Final RMSD
1ER8_E:I	3APR_E:I	0.917	PFHLLVY	PFHFV	1.57	0.84
1CKA_A:B	1PRM_C:A	0.750	PPPALPPKK	AFAPPLPRR	1.94	2.80
1AWR_C:I	1FGL_A:B	0.989	HAGPIA	VHAGPIAPGQR	1.44	1.37
1SFI_A:I	2BTC_E:I	0.996	GRCTKSIPPICFPD	RVCPKILMECKKDSD CLAECICLEHGYCG	2.13	2.81
1CZY_C:E	1QSC_A:D	0.926	PQQATDD	YPIQET	3.28	1.04
1DDV_A:B	1QC6_A:C	0.751	TPPSPF	EFPPPPT	8.16	7.23
1EG4_A:P	1BT6_A:C	0.617	NMTPYRSPPPYVP	STVHEILSKLS	51.13	42.96
1JBU_H:X	8GCH_G:C	0.875	EEWEVLCWTWETCER	GAW	31.12	26.03
1H6W_A:B	1FCH_A:C	0.150	SLNYIIKVKE	YQSKL	16.88	13.92
1KL3_C:G	1RST_B:P	0.911	HPQFEK	AWRHPQFGG	2.14	3.69
1GYB_B:E	1KL5_A:E	0.434	GFSFG	SHPQF	28.16	27.02
1LVM_A:E	1FN8_A:B	0.634	EATQLMN	GAR	25.20	26.44
1MFG_A:B	2PDZ_A:B	0.796	EYLGLDVPV	KESLV	6.04	2.68
1N7F_B:D	1BE9_A:B	0.683	ATVRTYSC	KQTSV	3.08	1.23
1OAI_A:B	1H27_B:E	0.176	DSGFSFGSK	RNLFGP	26.97	23.36
1NVR_A:B	1QMZ_A:E	0.737	ASVSA	HHASPRK	29.04	28.90
1NX1_A:C	1NPQ_A:B	0.621	DAIDALSSDFT	RMSADAMLRALLGSKHK	19.70	19.25
1OU8_B:D	3SEM_A:C	0.539	GAANDENY	PPPVPR	26.01	25.47
1UJ0_A:B	1OEB_B:C	0.850	TPMVNRENK	APSIDRSTKPPL	0.88	1.00
1RXZ_A:B	1ISQ_A:B	0.907	KSTQATLERWF	KQATLFDF	2.99	1.71
1SSH_A:B	3GBQ_A:B	0.823	GPPPAMPARPT	VPPPVPPRRR	2.16	1.38
1T4F_M:P	1YCR_A:B	0.801	RFMDYWEGL	ETFSDLWKLLPEN	1.14	1.30
1T7R_A:B	1T5Z_A:B	0.992	SSRFESLFAG	SEKFKLLFQSY	1.18	1.13
1VZQ_H:I	1GHW_H:I	0.995	DFEEIPE	DFEEIPEELQ	0.53	1.37
1TP5_A:B	1BE9_A:B	0.792	KKETWV	KQTSV	1.29	0.97
1W9E_A:T	1OBY_A:P	0.963	NEFYF	NEFYA	0.41	0.79
1YUC_A:C	1YOW_A:B	0.947	ASRPAILYALLSSS	AQALAALLAKA	2.37	2.03
1YWO_A:P	1SSH_A:B	0.811	QPPVPPQRPM	GPPPAMPARPT	3.75	3.54
1X2R_A:B	1P22_A:C	0.493	LDEETGEFL	YLDGIHGAT	11.27	8.18
2A3I_A:B	1KV6_A:C	0.926	QQKSLLQQLLTE	HKILHRLLQE	2.98	3.91
2AK5_B:D	2SEM_A:C	0.856	RPPKPRPR	PPPVPR	1.49	1.19
2B1Z_B:D	1X7E_A:C	0.894	KILHRLLQD	HKILHRLLQDS	0.40	4.78
2C3I_B:A	2BZK_B:A	0.970	KRRRHPSG	KRRRHPS	1.00	0.80
2B9H_A:C	1UKH_A:B	0.854	RRNLKGLNLNLH	PKRPTTLNLF	5.56	4.79
2FGR_A:B	1E54_A:B	0.990	DNWQNGTS	DNWQNGTS	0.99	1.49
2FMF_A:B	2FLW_A:B	0.968	QDQVDDLLDSLGF	SQDQVDDLLDSLGF	0.33	1.31
2FOJ_A:B	1YY6_A:B	0.949	GARAHSS	DPGEGPST	4.74	4.54
2CCH_D:F	1OKW_B:E	0.989	HTLKGRRLVFDN	RRLN	2.86	1.46
2FVJ_A:B	1ZGY_A:B	0.736	HKLVQLLTTT	PSILKKILLEEP	0.90	0.96
2H9M_C:D	2G9A_A:B	0.967	ARTKQ	ARTQ	0.63	0.94
2DS8_B:P	2FSA_A:P	0.186	ALRVVK	ARTQT	14.89	14.16
2HO2_A:B	1K9Q_A:B	0.565	PPPPPPPPL	GPPPY	14.05	16.73
2HPL_A:B	2AKA_A:L	0.095	DDLYG	TRLVPR	69.46	41.79
2O02_A:P	2C23_A:P	0.659	GHGQGLLDALDLAS	GLLDALDLASK	7.85	2.06
2O4J_A:C	1RKG_A:C	0.817	KNHPMLMNLLKD	NHPMLMNLLKD	0.67	0.91
2O9V_A:B	3GBQ_A:B	0.868	VPPPVPPPPS	VPPPVPPRRR	1.97	1.16
2P1K_A:C	1CMI_A:C	0.814	SATSAKATQTD	AEMKDTGIQVDR	2.11	3.31
2P1T_A:B	1XIU_A:E	0.741	HKILHRLLQD	HKILHRLLQEGS	0.76	1.32
2P54_A:B	1K7L_A:B	0.754	ARHKILHRLLQE	ERHKILHRLLQEG	1.78	1.98
2PUY_B:E	2G6Q_A:B	0.633	ARTKQTARKS	ARTQTAR	4.48	3.08
2QOS_C:A	1VWR_B:P	0.587	LRYDSTAERLY	CHPQGPPK	6.52	6.20

2R7G_C:D	1N4M_A:C	0.497	PPTLHELYDL	DDYLWGLEAGEGISDLFD	3.64	3.49
2VJ0_A:P	1KY6_A:P	0.949	PKGWVTFE	FSDPWGG	11.72	10.88
3BU3_A:B	2Z8C_A:B	0.371	YNPYPEDYGDIEIG	DYMNMS	3.54	4.44
2ZJD_A:B	2ASQ_A:B	0.735	GGDDDWTHLS	KVDVIDLTIESSSD	9.68	8.32
3D1E_A:P	1OK7_B:C	0.989	GQLGLF	RQLVLGL	1.79	1.58
3D9T_B:D	1XB1_A:G	0.604	ATPFQE	AVPIA	1.70	3.70
Average					8.60	7.57
Median					2.98	2.81

Table S4. Ligand-RMSD (LRMSD), interface-RMSD (IRMSD), fraction of native contact (fnat), and model quality measured by the CAPRI criterion (***/**/* for high-accuracy/medium-accuracy/acceptable prediction) for the predictions made on the 22 recently released targets by GalaxyPepDock and PEP-SiteFinder.

PDB_ID		GalaxyPepDock				PEP-SiteFinder			
Bound	Unbound	LRMSD	IRMSD	fnat	Quality	LRMSD	IRMSD	fnat	Quality
3FY2_A:B	4GK3_A	3.12	1.05	0.778	*	21.30	7.82	0.000	-
3G2S_A:C	1JWF_A	4.60	0.92	0.577	**	11.77	4.92	0.115	-
3GCI_A:P	1LN8_A	1.79	1.01	0.722	**	4.59	2.04	0.611	-
3GD1_I:Z	4G55_A	2.93	1.52	0.652	*	7.27	3.57	0.130	-
3GM1_A:E	3GM3_A	1.66	1.01	0.875	**	10.02	4.96	0.000	-
3I5R_A:B	1PKS_A	1.83	0.91	0.850	**	4.49	1.96	0.400	*
2WOK_A:B	2WOP_A	4.71	1.19	0.684	*	6.90	1.96	0.474	*
3IQQ_A:B	4FQO_A	2.85	1.71	0.480	*	4.49	2.41	0.280	-
3JZO_A:P	3LBJ_E	1.42	0.84	0.885	**	3.76	1.99	0.500	*
3O17_A:F	3PZE_A	1.46	2.09	0.826	**	15.86	7.62	0.174	-
2XXM_A:T	2JYL_A	1.31	2.87	0.467	*	2.70	1.60	0.300	*
3PTL_A:B	3DE2_X	13.37	6.02	0.154	-	5.53	2.50	0.231	-
3R42_A:B	3R3Q_A	27.07	11.45	0.000	-	11.34	6.79	0.130	-
3RL7_A:G	1ZOK_A	1.21	2.18	0.833	**	10.53	4.78	0.111	-
3SJ9_A:B	3SJ8_A	9.73	4.14	0.065	-	10.36	3.99	0.161	-
3TJV_A:B	3TK9_A	28.03	11.57	0.000	-	10.52	4.60	0.098	-
3UI2_A:B	3DEO_A	35.87	12.14	0.000	-	17.38	4.72	0.000	-
3V2X_A:B	3SO8_A	2.02	1.23	0.806	*	13.25	5.42	0.097	-
3VE6_A:B	1IAL_A	3.80	1.87	0.725	*	6.30	3.37	0.200	-
4F1Z_A:Q	4F24_A	2.08	1.96	0.776	*	21.29	9.39	0.034	-
4B8O_A:B	4B8J_A	1.49	1.02	0.703	**	10.06	5.08	0.162	-
4JDK_A:B	2X4Z_A	1.18	0.88	0.677	**	4.42	1.93	0.258	*
Average		6.98	3.16	0.570		9.73	4.25	0.203	
Median		2.46	1.61	0.693		10.04	4.29	0.162	

Table S5. Similarity of the query and the template protein structures measured by TM-score, sequences of the query and the template peptides and ligand RMSD of the starting complex and the final model on the 22 recently released complex structures.

ID (Query)	ID (Template)	TM-Score	Query peptide	Template peptide	Initial RMSD	Final RMSD
3FY2_A:B	2G2I_A:C	0.865	WDNYEFIW	EEIFGEFE	3.40	3.12
3G2S_A:C	1UJJ_A:C	0.965	GFSDDVPMVIA	DDISLLK	3.16	6.30
3GCI_A:P	1ZM6_A:P	0.984	VGGVVIA	LAIYS	4.57	1.79
3GD1_I:Z	1C9I_A:C	0.990	TNLIELDA	AVSLLDLDA	2.39	2.93
3GM1_A:E	1OW7_A:D	0.957	RELDELMASLS	TRELDELMASLS	1.62	1.66
3I5R_A:B	1NLP_C:N	0.777	KRPLPPLPS	PLPPLP	2.30	1.83
2WOK_A:B	1B5J_A:B	0.806	RPPGFR	KQK	5.62	4.71
3IQQ_A:B	1MWN_A:X	0.831	TKIDWNKIL	TRTKIDWNKILS	4.61	2.85
3JZO_A:P	3FE7_A:L	0.964	LTFEHYWAQLTS	FMWEL	0.44	1.42
3O17_A:F	2NO3_A:F	0.927	PKRPTTLNLF	PKRPTTLNLF	0.79	1.46
2XXM_A:T	3DS3_A:C	0.746	ITFEDLLDYYG	ITFEDLLDYYGP	0.75	1.31
3PTL_A:B	1BJR_E:I	0.996	KGEADALSLD	VAQGGAAGLA	13.29	13.37
3R42_A:B	1M4Q_A:B	0.734	QVPSDPYN	PEPTAPPEE	28.05	27.07
3RL7_A:G	2I0I_A:D	0.731	SYLVTSV	RRETQV	1.84	1.21
3SJ9_A:B	2H9H_A:I	0.726	GLRQAVTQ	LAA	10.76	9.73
3TJV_A:B	2HWL_D:P	0.912	PTSYAGDDS	PAETEDSLPEDD	28.64	28.03
3UI2_A:B	3DEP_A:B	0.936	QKAPPGTARRKRK	YPGGSFDPLGLA	37.53	35.87
3V2X_A:B	3UXG_A:B	0.938	LLPTLPKLPSL	PLYTSPSLPNITLGLP	2.16	2.02
3VE6_A:B	1EE4_A:C	0.948	EGPSAKKPKKEA	PAAKRVKLD	3.90	3.80
4F1Z_A:Q	3AT0_A:B	0.982	YGGGSSGGGSSGGG	WNSGSSGTGSTG	3.03	2.08
4B8O_A:B	1EE4_A:C	0.947	GSPPKKKRKVG	PAAKRVKLD	1.74	1.49
4JDK_A:B	2Q0N_A:B	0.945	RRRSWY	RRRRRSWYFDG	1.51	1.18
Average					7.37	7.06
Median					3.10	2.46

Table S6. Prediction made by GalaxyPepDock on the CAPRI target 67 compared with those submitted by top 3 servers and top 6 human groups in the CAPRI blind prediction experiment.

	LRMSD	IRMSD	fnat	Quality
GalaxyPepDock	1.80	1.01	0.688	**
**Server Predictors**				
SwarmDocK	2.92	1.37	0.625	*
HADDOCK	3.18	1.94	0.500	*
ClusPro	4.18	1.49	0.688	*
**Human Predictors**				
Bates	1.12	0.80	0.688	**
Furman	1.27	0.93	0.938	**
Zhou	1.40	1.11	0.688	**
Niv	1.43	0.99	0.688	**
Zacharias	1.62	0.80	0.875	**
Vajda	1.69	1.23	1.000	**
